# The level of socioeconomic development of EU countries and the state of ISO 14001 certification

**DOI:** 10.1007/s11135-015-0297-7

**Published:** 2015-12-21

**Authors:** Barbara Fura, Qingfang Wang

**Affiliations:** 1 Faculty of Economics, University of Rzeszów, Ćwiklińskiej 2, 35-601 Rzeszów, Poland; 2 School of Public Policy, University of California Riverside, Riverside, CA 92521 USA

**Keywords:** Environmental management, ISO 14001 standard, Socioeconomic development, Hellwig’s synthetic indicator, European Union

## Abstract

This study examines the relationship between the level of socioeconomic development of the EU 28 countries and the adoption of International Organization for Standardization (ISO) 14001 environmental management system. First, through a multivariate comparative analysis of the secondary data obtained from the public statistics, a Hellwig’s synthetic indicator is created to rank the level of socioeconomic development of the EU 28 countries. Then, using the total number of certificates issued in 2012 and the increase from 2011, this study has found a correlation between the level of national socioeconomic development and the adoption of ISO 14001 system in their businesses. Although there was no relationship between the number of ISO 14001 certificates in 2012 and the level of socioeconomic development at the national level, a weak negative correlation was observed between the increase of certification from 2011 to 2012 and the level of national socioeconomic development. The results suggest a higher interest in ISO 14001 adoption by the firms from the less developed countries than those from the more developed states.

## Introduction

The changing business environment and intensified competition have urged many companies to operate their businesses in compliance with accepted norms and standards which include not only general regulations of business activities, but also social norms and standards in the area of environmental protection (Chen and Chang 2013; Hąbek and Wolniak 2015). Commonly binding principles, norms and standards can bring greater predictability and thus reduced risks for businesses. It is specifically important for countries as members of international economic institutions and organizations, including countries newly admitted into the EU as well as those looking forward to accession. Yet, requirements for standardisation are relatively new issues that pose more challenges for companies from the less developed states.

Since the mid-1990s, various voluntary actions in environmental management have been adopted by firms around the world. One of the most notable practice is the adoption of International Organization for Standardization (ISO) 14001 standard (He et al. 2015; Hąbek 2014) developed by ISO, a non-governmental body located in Geneva, Switzerland. The ISO 14000 series of standards was based on the need expressed at the United Nations Conference on Environment and Development in Rio de Janeiro in 1992. The main aim of the new series of standards was to encourage businesses to systematically improve environmental quality (Bansal and Bogner 2002).

ISO 14001 is the only standard designed for the purpose of audit and certification in the ISO 14000 series. The core elements of the ISO 14001 standard include environmental policy, planning, implementation and operation, checking and corrective action, review, and improvement (Franchetti 2011). The ISO 14001 system supposes to be a worldwide solution, applicable in any organization who is interested in managing its environmental impacts for continuous improvements. The strength of this international standard lies in its flexibility to maintain the internal structures and prevailing features of the businesses while executing the most effective means to improve environmental impacts (Testa et al. 2014). Overall, the compliance of environmental management systems with ISO 14001 enables firms to identify and control their environmental impacts, improve their environmental performance continually, and implement a systematic approach to achieve environmental goals (McGuire 2014).

This is why the adoption of ISO 14001 can bring numerous internal and external benefits (e.g., Fura 2013; He et al. 2015; Nishitani 2010; Testa et al. 2014; To and Lee 2014; Zobel 2013), such as cost reduction, energy saving, improvement in environmental performance, process and product innovations, efficiency improvement, corporate image improvement, entering new markets, increase in market share, and reduction in insurance fees. Turk (2009) listed four aspects of benefits for ISO 14001 certified businesses: environmental benefits and internal operation, corporate management, marketing effects, and subcontractor relations. Similarly, Gavronski et al. (2008) argued that businesses will gain in productivity, financial, societal and market benefits.

Businesses in different countries, regions and industries are widely interested in the adoption of ISO 14001 (Qi et al. 2011). Although ISO 14001 compliance is not legally enforced, the total number of ISO 14001 adoptions in the world has steadily increased since the release of the system in 1996 (Lagodimos et al. 2007), reaching 301,647 certifications by 2013 (The ISO Survey 2013). 39.3 % of the issued certificates in 2012 were for organizations located in European countries and the share of Europe remained the same in 2013 (39.5 %). In 2012 the worldwide increase in the total number of ISO 14001 certificates was 9 %, and the annual growth rate decreased to 6 % in 2013. The increase in Europe was 11 % in 2012, and the rate decreased to 6 % in 2013 as well (The ISO Survey 2012, 2013).

Despite the positive effect of ISO 14001 adoption, the range and the scale of its benefits might differ among the businesses, depending on the business characteristics as well as their environments. At the global level, some studies have analyzed the effects of various economic, market, and regulatory factors on the adoption of ISO 14001 certification. For example, Neumayer and Perkins (2004) found that the number of ISO 14001 certificate is positively correlated with the income of the country, foreign direct investments, and exportation. In the same vein, Fikru (2014) showed that businesses that export, have some foreign ownership and are internationally connected have a higher probability of certifying.

Scholars around the world have examined the types of businesses adopting the ISO 14001 environmental management system. For example, Chapple et al. (2001) showed that among large manufacturing companies in the UK, the adoption of ISO 14001 system is related to the companies’ market position. According to them, when their market position is stronger, the pressure to adopt the ISO 14001 becomes lower for these businesses. Other studies in Japan showed that companies are more willing to implement such systems if their activities are based on less complex processes, which suggests that businesses involved in high-risk activities are more likely to implement such systems (Takahashi and Nakamura 2010). To and Lee (2014) examined the dependency between the number of ISO 14001 certificate and export volume in the top 30 countries. They found that the relationship between the two factors weakens when the number of ISO 14001 certificate saturates. At the same time, some researchers argue that there is no connection between the adoption of ISO 14001 standard and the type of business or business location (e.g., Marimon et al. 2011).

Despite accumulation of the literature on the relationship between ISO 14001 adoption and business characteristics, knowledge is still very limited about the adoption of ISO 14001 systems and the external factors of business activity, such as the level of national development in the countries where the businesses are located. Therefore, the objective of the current study is to examine the relationship between the level of socioeconomic development of the EU member states and their adoption of ISO 14001 standard. Based on the above discussion of existing studies, we hypothesize that:

### **H**

Organizations located in less developed countries of EU are more likely to adopt ISO 14001 system than firms from more developed countries.

## Methodology

### Data source

The empirical analysis in this study was based on currently available statistical sources, i.e., Eurostat, EU economics—economic indicators platform, Central Statistical Office of Poland publications. Data on the ISO 14001 certification in the EU 28 member states were obtained from the official publications of ISO (*The ISO Survey of Certifications* 2012, 2013).

### Analysis

Measuring socioeconomic development is highly complex due to the wide spectrum of covered factors (Grzebyk and Stec 2015). Thus, to examine the EU countries’ development level we apply a multivariate comparative analysis, i.e., linear ordering of objects. Originally this method was created by Hellwig (1968) which allows to create a ranking of objects measured by multiple variables. Through this method, objects in study are ordered on the basis of their distance from the established reference object.

We first select diagnostic variables to create a Hellwig’s synthetic indicator, based on these variables’ significance, the level of variability, and the level of correlation of variable pairs. The statistically formal criteria of the applied method concerns an appropriate level of variation of the features and a low correlation between the variables (Guyon and Elisseeff 2003). To examine the level of variation we apply the classical coefficient as provided below:

1 $$v_{j} = \frac{{S_{{x_{j} }} }}{{\bar{x}_{j} }} \cdot 100,$$

where *v*_*j*_ is the coefficient of variation, $S_{{x_{j} }}$ is the standard deviation of the *j*th variable, and $\bar{x}_{j}$ is the mean value of *j*th variable. We take 10 % critical value for the coefficient of variation (Perło 2014). This means that variables with the coefficient of variation less than or equal to 0.1 are considered as quasi-stable (i.e., devoid of enough information load) and thus are eliminated from the further analysis.

To measure the correlation between the variables we use Pearson’s correlation coefficient given by (2):

2 $$r_{xy} = r_{yx} = \frac{{\tfrac{1}{n}\sum\nolimits_{i = 1}^{n} {(x_{i} - \bar{x})(y_{i} - \bar{y})} }}{{S_{x} S_{y} }} = \frac{{\text{cov} (x,\;y)}}{{S_{x} S_{y} }},$$

where *r*_*xy*_ is the coefficient of correlation, *S*_*x*_ and *S*_*y*_ are standard deviations of the *x*th and *y*th variables, and *n* is the number of variables. The critical value of the correlation coefficient is the absolute value of ±0.7 (Stec et al. 2014). Based on this criterion, we selected the variables for analyses whose coefficient of correlation with other variables is lower than this threshold value.

Different characteristics of each country have different units. To eliminate the problems with different units, we standardize their values through the following calculation (Gostowski 1970):

3 $$z_{ij} = \frac{{x_{ij} - \bar{x}_{j} }}{{S_{{x_{j} }} }},$$

where *i* is the number of objects (countries), *j* is the number of variables, $\bar{x}_{j}$ is the mean value of *j*th variable, $S_{{x_{j} }}$ is the standard deviation of the *j*th variable, and *z*_*ij*_ is the standardized value of the *j*th variable for the *i*th object. We use these standardized values to establish the pattern of development, i.e., the “ideal object” with the coordinates: *z*_01_, *z*_02_,*…*,*z*_0*k*_, where *z*_0*j*_ = *max*_*i*_{*z*_*ij*_} represents variables that are stimulants; and *z*_0*j*_ = *min*_*i*_{*z*_*ij*_} represents variables that are destimulants.^1^

Then, for each object we calculate its distance from the “ideal object” by the following formula (Lesot 2006):

4 $$d_{i} = 1 - \frac{{D_{i0} }}{{D_{0} }},\quad (i = 1, \ldots ,n),$$

where *d*_*i*_ is the measure of development proposed by Hellwig, *D*_*i*0_ is the Euclidean distance between the country and the “ideal object”, and *D*_0_ is the critical distance between the objects and the “ideal object”. The calculation of the Euclidean distance (*D*_*i*0_) is based on the following formulas:

5 $$D_{i0} = \sqrt {\sum\limits_{j = 1}^{m} {\left( {z_{ij} - z_{0j} } \right)^{2} } } ,$$

6 $$D_{0} = \overline{{D_{0} }} + 2S_{0} ,$$

7 $$\overline{{D_{0} }} = \frac{1}{n}\sum\limits_{i = 1}^{n} {D_{i0} } ,$$

8 $$S_{0} = \sqrt {\frac{1}{n}\sum\limits_{j = 1}^{m} {\left( {D_{i0} - \overline{{D_{0} }} } \right)}^{2} } .$$

The synthetic indicator calculated for each object (an EU country) is assumed to take a positive value that ranges from 0 to 1 (Ostasiewicz 1998). The closer the value of a given object is to the “ideal object”, the higher the level of development.

Moreover, to simplify a comparison among countries’ development level we apply a modification of the indicator according to the following formula (Sarama 2013):

9 $$d_{i}^{\prime} = 100 \cdot \frac{{d_{i} }}{{\hbox{max} \{ d\} }},$$

where *max*{*d*} is the maximum value of the synthetic variable. Thus, the transformed indicator (*d*′) for the most developed country takes a value of 100.

We also use the value of the indicator (*d*′) to create groups of objects with similar levels of socioeconomic development. In order to establish the class limits, we apply the arithmetic mean of the indicator ($\overline{{d^{\prime} }}$) and the value of a standard deviation (*s*), thus providing the following classification:

10 $$\begin{aligned} &{\text{Group}}\;{\text{I}}\;{\text{contains}}\;{\text{countries}}\;{\text{with}}\;{\text{a}}\;{\text{high}}\;{\text{level}}\;{\text{of}}\;{\text{development:}}\;d_{i}^{\prime} > \overline{{d^{\prime} }} + s; \hfill \\ &{\text{Group}}\;{\text{II}}\;{\text{contains}}\;{\text{countries}}\;{\text{with}}\;{\text{a}}\;{\text{level}}\;{\text{of}}\;{\text{development}}\;{\text{above}}\;{\text{average:}}\;\overline{{d^{\prime} }} < d_{i}^{\prime} \le \overline{{d^{\prime} }} + s; \hfill \\ &{\text{Group}}\;{\text{III}}\;{\text{contains}}\;{\text{countries}}\;{\text{with}}\;{\text{a}}\;{\text{level}}\;{\text{of}}\;{\text{development}}\;{\text{below}}\;{\text{average:}}\;\overline{{d^{\prime} }} - s < d_{i}^{\prime} \le \overline{{d^{\prime} }} ; \hfill \\ &{\text{Group}}\;{\text{IV}}\;{\text{contains}}\;{\text{countries}}\;{\text{with}}\;{\text{a}}\;{\text{lowest}}\;{\text{level}}\;{\text{of}}\;{\text{development:}}\;d_{i}^{\prime} \le \overline{{d^{\prime} }} - s. \hfill \\ \end{aligned}$$

To examine the relationship between the development level of the EU countries and ISO 14001 certification, we employ Pearson’s correlation coefficient given by (2). The level of development of the countries is characterized by abovementioned Hellwig indicator. The absolute numbers of ISO 14001 certificate in the countries are difficult to compare with each other since they do not reflect the countries’ characteristics (Casadesús et al. 2008). Thus, we introduce the relative measure of ISO 14001 certification through dividing the ISO 14001 certificate number in 2012 and its increase from 2011 to 2012 by countries’ total population in 2012. Although both variables are in relative forms, it is still possible to use Pearson’s correlation analysis to measure the relationship between the ISO adoption and national socioeconomic development.

### Variables

Socioeconomic development in a country is a multi-dimensional process influenced by many factors. Measuring the level of development thus requires choosing appropriate descriptive variables. This study used a set of 15 variables of national socioeconomic development to cover the areas of economy, environment and society (Borys 2010; Išljamović et al. 2015). Specifically, we employed the following indicators:

- X_1_: GDP per capita (USD),
- X_2_: rate of inflation (%),
- X_3_: rate of unemployment (%),
- X_4_: changes in industrial production (%, 2012–2011),
- X_5_: energy intensity of the economy (kg oil equivalent/1000 EUR),
- X_6_: employment rate (%),
- X_7_: resource efficiency [GDP/domestic material consumption (DMC)],
- X_8_: percentage of people at risk of poverty/exclusion (%),
- X_9_: export/import indicator (%),
- X_10_: participation of hi-tech exports in exports (%),
- X_11_: rate of poverty risk/exclusion for people at the age 25–29 (%),
- X_12_: lifelong learning participation by educational attainment (% of people aged 25–64 benefiting from education and trainings),
- X_13_: percentage of population who has never used Internet (% of population aged 16–74),
- X_14_: proportion of expenditures on R&D in GDP (%),
- X_15_: inflow of FDI (% of GDP).

To measure the national economic development, we use traditional indicators such as GDP per capita, rate of inflation, rate of unemployment (employment) or export/import indicator. The first indicator (X_1_) is to compare the standard of living in the countries. We examine the stability of the economies as well as their market security with the use of rate of inflation (X_2_). X_3_ and X_6_ reflect the labor market situation and the changes in industrial production (X_4_) indicates the economic activity in the member states. The position of the countries on the international trade is reflected by the export–import ratio (X_9_). The share of hi-tech exports (X_10_) represents the level of the technological advancement. The proportion of expenditures on R&D in GDP (X_14_) examines the innovativeness of the countries. Essential for its improvement especially in less developed countries might be also the inflow of foreign direct investment reflected by X_15_.

In the area of environmental aspects of national development, we consider the energy intensity of the economy (X_5_) and resource efficiency (X_7_). The first indicator measures the energy consumption and its overall energy efficiency. The second one reflects the productivity of raw materials such as fuels, minerals, metals, as well as food, soil, water, air, biomass, and ecosystems.

The rest of the indicators deal with social issues of the development. The first area of interest is the problem of poverty reflected by poverty risk examined in general (X_8_) and among the youth (X_11_). The second issue is the education level of society expressed by the interest in lifelong education among the 25–64 age group (X_12_). The third one is the level of Internet usage as measured by the share of non-users among the 16–74 age group (X_13_). We also treat this indicator as the proxy of standard of living.

The descriptive statistics for X_1_–X_15_ variables are provided by Table 1, followed by the detailed discussion.

**Table 1:** Descriptive statistics

Variables	X_1_	X_2_	X_3_	X_4_	X_5_	X_6_	X_7_
Average	30,968.5	2.9	10.5	99.2	222.3	67.9	133.8
Minimum	7043.0	0.9	4.3	93.1	82.8	55.3	62.0
Maximum	107,206.0	5.7	25.0	108.0	669.9	79.4	214.3
Standard deviation	20,374.7	0.9	5.2	3.7	130.6	6.3	31.6
Coefficient of variety (%)	65.8	31.7	49.1	3.7	58.8	9.3	23.7

Variables	X_8_	X_9_	X_10_	X_11_	X_12_	X_13_	X_14_	${\text{X}}_{15}^{\text{a}}$
Average	25.6	1.0	11.6	25.7	10.0	23.2	1.7	1.8
Minimum	15.0	0.9	3.2	13.4	1.4	5.0	0.5	−1.2
Maximum	49.3	1.3	31.8	44.2	31.6	48.0	3.6	8.9
Standard deviation	8.2	0.1	7.1	8.4	7.5	12.0	0.9	2.7
Coefficient of variety (%)	32.2	9.4	61.2	32.8	74.8	51.7	55.2	149

*Source* based on http://epp.eurostat.ec.europa.eu/portal/page/portal/eurostat/home/; http://euro-dane.com.pl; Sytuacja społeczno-gospodarcza w Unii Europejskiej w *r.* (2013), http://www.stat.gov.pl/gus/5840_11534_PLK_HTML.htm

^a^Except Luxembourg

#### Economy

The highest level of GDP per capita was observed in 2012 in Luxembourg, followed by Sweden, Austria and the Netherlands. On the other end, Bulgaria and Romania had the lowest GDP per capita. In the same measurement, Poland ranked the 25th among the EU 28 countries, surpassed by such countries as the Czech Republic, Slovakia, Estonia, Latvia, Lithuania, and Hungary. The highest rate of unemployment was in Spain (25 %), followed by Greece (24.3 %) and Croatia and Portugal (15.9 %). The rate of unemployment in Poland was 10.1 %, similar to Estonia, France, Italy, and Hungary. The rate of employment in Poland was lower than the EU average by about 5.0 %. The highest employment rate was observed in Sweden (79.4 %) and the lowest in Greece (55.3 %). At the same time, Poland had a relatively higher rate of inflation in 2012, only lower than Hungary (5.7 %) and Estonia (4.2 %).

The highest market openness, measured as the ratio of export–import, was observed in Ireland (1.29), with the lowest in Greece (0.85). The value of this indicator for Poland (1.01) was lower than the average by about 2.7 %. The proportion of exports of hi-tech products in overall exports was 5.9 % in Poland, ranked 22nd in the EU. The highest values of this indicator was observed in Malta (31.8 %), followed by Luxembourg (26.2 %) and Ireland (20.6 %), and the lowest value in Portugal (3.2 %), Greece (3.3 %), and Bulgaria (3.8 %).

Not surprisingly, the percentage of expenditures on R&D in GDP was pretty low in Poland (0.9 %), ranked 20th of the EU. In comparison, this rate was the highest in Finland (3.6 %) and lowest in Cyprus (0.5 %). For the inflow of FDI as percentage of GDP, the highest value was noted in Luxembourg (486.5 %), 54 times higher than that in Hungary (8.9 %), the second-highest country. Due to the extremely high dispersion of this variable across the countries, the descriptive statistics of X_15_ presented in Table 1 excluded Luxembourg. For this indicator, Poland ranked 21st with a value of 0.1 %. Further, an increase in industrial production, in comparison to 2011, was observed in Hungary, Malta, Latvia, Lithuania, Romania, Poland and Estonia. The industrial output decreased from 2011 to 2012 in all the other countries.

#### Environment

The economy with the lowest level of energy intensity was Ireland, and the highest was Bulgaria. The value of this indicator for Poland was about 34 %, higher than the EU average. Polish economy was also characterized by a lower than average (about 6 %) resource efficiency. The highest value of that indicator (in kg oil equivalent per 1000 EUR) was observed in Ireland (82.8), and the lowest in Romania (669.9).

#### Society

In Poland, 26.7 % of the total population were at risk of poverty or social exclusion, slightly higher than the EU average (25.6 %), while the highest value of that indicator was observed in Bulgaria (49.3 %) and the lowest in the Netherlands (15 %). However, the economic situation of the young population (25–29 years old) in Poland was more promising. The poverty risk of this age group was 21 %, lower than the EU average by 18 %. The percentage of people attending in lifelong learning programs in Poland was only 4.5 %, twice lower than the EU average. Such a rate was highest in Denmark (31.6 %), followed by Sweden (26.7 %) and Finland (24.5 %), and lowest in Romania (1.4 %). In Poland, 32 % of total population aged 16–74 have never used Internet, and this rate is lower than that in Portugal, Croatia, Cyprus, Italy, Bulgaria, Greece, and Romania.

Next, we assessed the level of variation in these variables. As mentioned in Sect. 2.2, the coefficient of variation is expected to exceed 10 %. This condition was not satisfied in case of X_4_: change in industrial production, X_6_: rate of employment, and X_9_: export/import rate. Thus, these features were excluded.

Another criterion for variable selection was the level of correlation among variables which is shown by Table 2. Based on the threshold values of ±0.7 (Stec et al. 2014), the following variables were further removed: X_8_: percentage of people at risk of poverty/exclusion, X_12_: lifelong learning participation by educational attainment, X_13_: percentage of population who has never used Internet; and X_15_: inflow of FDI (% GDP).

**Table 2:** Pearson’s correlation coefficient

Variables	X_1_	X_2_	X_3_	X_5_	X_7_	X_8_	X_10_	X_11_	X_12_	X_13_	X_14_	X_15_
X_1_	1.00											
X_2_	−0.35	1.00										
X_3_	−0.34	−0.21	1.00									
X_5_	−0.58	0.34	0.02	1.00								
X_7_	0.12	−0.05	0.36	−0.32	1.00							
X_8_	−0.58	0.07	0.43	0.54	−0.07	1.00						
X_10_	0.53	0.12	−0.44	−0.30	0.07	−0.42	1.00					
X_11_	−0.30	−0.20	0.41	0.15	0.05	0.77	−0.43	1.00				
X_12_	0.50	−0.27	−0.33	−0.39	−0.04	−0.64	0.12	−0.26	1.00			
X_13_	−0.70	0.15	0.44	0.41	−0.11	0.79	−0.49	0.57	−0.71	1.00		
X_14_	0.49	−0.25	−0.43	−0.37	−0.02	−0.72	0.14	−0.34	0.77	−0.74	1.00	
X_15_	0.73	0.01	−0.21	−0.13	0.03	−0.17	0.40	−0.17	0.11	−0.29	−0.03	1.00

Thus, the final accepted indicators include: X_1_, value of GDP per capita (USD); X_2_, rate of inflation; X_3_, rate of unemployment; X_5_, energy intensity of the economy (kg of oil equivalent/1000 EUR); X_7_, resource efficiency (GDP/DMC); X_10_, participation of hi-tech exports; X_11_, rate of poverty/exclusion of people aged 25–29; and X_14_, expenditures on R&D in GDP.

The separate analysis of each variable cannot reflect the multi-dimensions and complexity of national socioeconomic development. As discussed earlier (Table 1), some countries may rank pretty high by certain indicators, but not necessarily by others. Therefore, in order to capture the overall level of development, we further apply a multivariate analysis to produce a synthesized indicator. In this step, we divide the features into stimulants and destimulants. The set of stimulants include the following features:

11 $${\text{S:}}\;\left\{ {{\text{X}}_{1} ,\;{\text{X}}_{7} ,\;{\text{X}}_{10} ,\;{\text{X}}_{14} } \right\},$$

while the set of destimulants include the following:

12 $${\text{D:}}\;\left\{ {{\text{X}}_{2} ,\;{\text{X}}_{3} ,\;{\text{X}}_{5} ,\;{\text{X}}_{11} } \right\}.$$

Finally, we use Formula (3) to standardize the features’ values based on which we use Formulas (4)–(8) to obtain the ranking of the EU counties.

## Results and discussion

### Ranking of EU countries

The level of overall socioeconomic development is represented by Table 3. Luxemburg, France and Sweden who belong to the old EU member states (EU 15) top the ranking. Above average are also the other members of the EU 15, except for Italy, Portugal, Spain and Greece who are classified below average. Among those which entered EU in 2004, the level of socioeconomic development is above the average in the following countries (from the top): Slovenia, the Czech Republic, Malta, and Cyprus. In the same 2004 EU country group, the development in Lithuania, Slovakia, Latvia, Poland, Estonia, and Hungary are below the average. Croatia, the newest member state of EU (entered EU in 2013), also ranks lower in the development hierarchy, followed by Romania and Bulgaria which joined EU in 2007.

**Table 3:** Level of socioeconomic development in the EU countries

Rating	EU 28	di	di′	Rating	EU 28	di	di′
1	Luxembourg	0.86	100.00	15	Cyprus	0.63	73.27
2	France	0.83	96.36	16	Italy	0.62	72.07
3	Sweden	0.82	96.29	17	Portugal	0.58	67.88
4	Netherlands	0.82	95.49	18	Lithuania	0.57	67.11
5	Germany	0.80	93.40	19	Slovakia	0.54	63.29
6	Austria	0.80	93.25	20	Latvia	0.54	62.86
7	UK	0.79	92.62	21	Poland	0.53	62.33
8	Ireland	0.78	90.90	22	Spain	0.51	59.08
9	Belgium	0.74	86.62	23	Estonia	0.48	55.48
10	Finland	0.74	86.08	24	Hungary	0.47	55.31
11	Slovenia	0.74	86.04	25	Croatia	0.46	54.24
12	Czech Republic	0.70	82.23	26	Greece	0.34	39.50
13	Denmark	0.70	81.72	27	Romania	0.29	34.22
14	Malta	0.69	80.60	28	Bulgaria	0.17	19.68

Differentiation in the countries’ development level is confirmed by the coefficient of variation (27.84 %). Moreover, a weak left-handed asymmetry (*As* = −0.81) of indicator’s values shows preponderance of countries developed better than the average. Above the average were classified 15 from 28 countries. However, the development level of the first in the ranking, Luxemburg, is more than five times higher than that in the last, Bulgaria. This difference indicates extreme unevenness in socioeconomic development among the EU.

### Grouping of EU countries

**Fig. 1:**
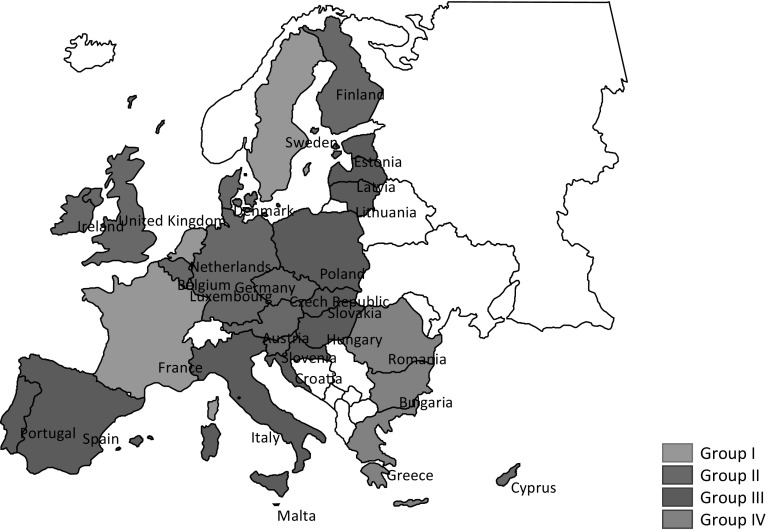
Classification of the EU 28 countries by the level of socioeconomic development

Based on the synthetic indicator of national socioeconomic development, the EU 28 member states are classified into four groups, as shown in Table 4 and Fig. 1.

**Table 4:** Classification of the EU member states by their level of development

Groups	Ranges	EU 28 countries
I	$d_{i}^{\prime}$ > 93.50	Luxembourg, France, Sweden, Netherlands
II	73.14 < $d_{i}^{\prime}$ ≤ 93.50	Germany, Austria, UK, Ireland, Belgium, Finland, Slovenia, Czech Republic, Denmark, Malta, Cyprus
III	52.78 < $d_{i}^{\prime}$ ≤ 73.14	Italy, Portugal, Lithuania, Slovakia, Latvia, Poland, Spain, Estonia, Hungary, Croatia
IV	$d_{i}^{\prime}$ ≤ 52.78	Greece, Romania, Bulgaria

The overall classification of socioeconomic development for the EU 28 member states is also mapped in Fig. 1.

The first group of highly developed countries were characterized with high GDP per capita, high standard of living, a relatively low level of unemployment (except for France where the unemployment rate was 10.2 %). The overall pattern indicates a stable labour market and minor risks of poverty or social exclusion, in particular among the youth. These economies have grown rapidly in the development of new technologies, supported by high expenditures on R&D. The significantly high proportion of hi-tech products in their exports confirms the innovativeness of these countries.

The second group, countries with the level of development higher than the average but lower than the first group, included Germany, Austria, UK, Ireland, Belgium, Finland, Slovenia, the Czech Republic, Denmark, Malta, and Cyprus. These are countries with well-developed economies, stable socioeconomic situations, high living standards, as well as environment-friendly economies characterized by low emissions. Malta and Cyprus ranked particularly high in this group, 14th and 15th, respectively. Malta was characterized by its high level of innovativeness in exports (31.8 %) and low risk of poverty and exclusion amongst the youth (15.1 %), as well as low unemployment rate (6.4 %). Cyprus had a relatively high share of population aged 24–64 participating in lifelong learning programs, as well as its innovativeness in exports (11.7 %).

Lower than the average level of socioeconomic development (Group III) was noted in Italy, Portugal, Lithuania, Slovakia, Latvia, Poland, Spain, Estonia, Hungary, and Croatia. The GDP per capita was lower than average in all these countries except for Italy. The rate of employment was also lower than the EU 28 average except for Estonia and the rate of unemployment in the third group (with the exception of Estonia and Lithuania) was higher than the EU average. Their living standards were also lower when compared to the first and second groups. The situation of this group was further deteriorated by the poor participation in lifelong education among its population. Moreover, there was a high proportion of people aged 16–74 who had never used the Internet. Further, lower expenditures on R&D further took away the opportunities for innovations and development of new technologies there. Poland was rated 21st, ahead of Spain, Estonia, Hungary and Croatia from the same group. The unemployment rate, the growth rate of industrial production, and the exports–imports ratio for Poland were slightly better than the EU average. However, values for all the other indicators pointed to a worse than the average socioeconomic situation of Poland.

Worst of all member states, Greece, Romania and Bulgaria were among the countries with the lowest level of socioeconomic development (Group IV). Greece, which was the most severely affected by the economic crisis of recent years, exhibited one of the highest rate of unemployment amongst the EU countries. The unfavourable situation of the fourth group countries was further reflected by the highest percentage of people aged 25–64 not attending lifelong learning, the highest percentage of people not using Internet, and the significantly high risk of poverty among the young and the total population. The weak socioeconomic situation of Bulgaria and Romania was also reflected by the high energy intensity of the economy, which was over three times higher in Bulgaria and almost twice higher in Romania than the EU average. Moreover, their extremely limited expenditures on R&D had significantly impeded their economic growth and widened their gap of development with the most developed states, such as Luxemburg, France, Sweden or the Netherlands.

### Hypothesis verification

As shown in Table 5, in 2011, the top three countries within EU with the largest number of ISO 14001 certificates were Italy (17,418), Spain (16,341) and UK (15,231). Ranked at 10th, Poland had 1900 certificates, after the Czech Republic (4451) but ahead of Slovakia (1152). Measured by national development, both of these two Central-Eastern Europe countries ranked higher than Poland (Table 3).

**Table 5:** Number of ISO 14001 certificates and its increase in the EU countries

EU countries	ISO 14001 (2011)	ISO 14001 (2012)	ΔN = N_2012_ − N_2011_	EU countries	ISO 14001 (2011)	ISO 14001 (2012)	ΔN = N_2012_ − N_2011_
Luxembourg	28	49	21	Cyprus	107	32	−75
France	7771	7975	204	Italy	17,418	19,705	2287
Sweden	4049	3885	−164	Portugal	836	1184	348
Netherlands	2489	2085	404	Lithuania	703	680	−23
Germany	7814	7034	780	Slovakia	1152	1426	274
Austria	963	1084	121	Latvia	250	237	−13
UK	15,231	15,884	653	Poland	1900	2014	114
Ireland	663	417	−246	Spain	16,341	19,470	3129
Belgium	727	1026	299	Estonia	358	394	36
Finland	1169	1310	141	Hungary	1580	1718	138
Slovenia	414	420	6	Croatia	488	760	272
Czech Republic	4451	4215	−236	Greece	543	657	114
Denmark	994	1756	762	Romania	7394	8633	1239
Malta	18	23	5	Bulgaria	927	1395	468

*Source* based on *ISO Survey of Certification* (2012, 2013), http://www.iso.org/iso/iso-survey

The top three countries with the largest number of the ISO 14001 certificates in 2012 were still the same: Italy (19,705), Spain (19,470) and UK (15,884). Poland still ranked 10th, with the total number of certificates increased to 2014. The highest increase from 2011 to 2012 in the total number of the ISO 14001 certificates was in Denmark (43.4 %), Croatia (35.8 %), and Bulgaria (33.5 %). The annual increase for Poland was 6 %. A decrease in the number of ISO 14001 certificates was noted in Cyprus (70 %), Ireland (37.1 %), the Czech Republic (5.3 %), Latvia (5.2 %), Sweden (4.1 %), and Lithuania (3.3 %).

**Fig. 2:**
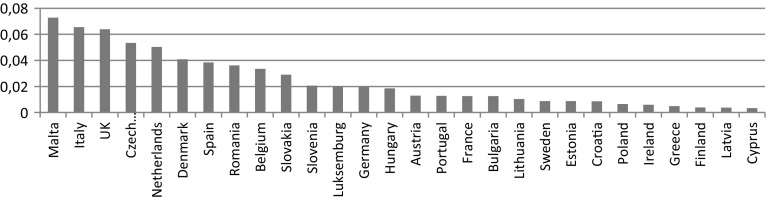
ISO 14001 certificates per capita in the EU countries (2012)

If considering the population size in each country (Fig. 2), the top five countries in the number of certificates per capita were Malta, Italy, UK, the Czech Republic, and the Netherlands. Poland ranked 23rd, followed by Ireland, Greece, Finland, Latvia, and Cyprus.

**Fig. 3:**
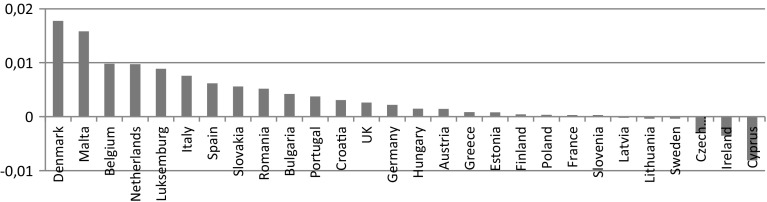
Change in ISO 14001 certificates number per capita in the EU countries (from 2011 to 2012)

The highest relative increase in ISO 14001 was observed in Denmark, Malta, Belgium, Netherlands, and Luxemburg (Fig. 3). The decrease was however observed in six countries: Cyprus, Ireland, the Czech Republic, Sweden, Lithuania, and Latvia. Poland observed an increase in ISO 14001 number per capita, but, the level of growth was one of the lowest, comparable to Finland, France or Slovenia.

Table 6 presents Pearson’s correlation results on the relationship between ISO 14001 certification adoption and the EU countries’ development. Between the number of ISO 14001 certificates and the synthetic indicator of socioeconomic development we observed a very weak negative correlation (−0.16). A stronger correlation (−0.29), however, was observed between the growth of certificates (from 2011 to 2012) and the level of development. The negative sign of the both coefficients of correlation shows that the adoption of ISO 14001 standard was more likely to be observed in countries with a lower level of socioeconomic development, rather than in more developed countries.

**Table 6:** Pearson’s correlation results

Pair of variables	r
ISO 14001 certificates per capita and EU national development	−0.1573
Increase of ISO 14001 certificates per capita and EU national development	−0.2913

## Discussion and conclusion

The assessment of national socioeconomic development is exceptionally difficult, due to the complexity of the phenomenon and the difficulty in measuring the diagnostic variables. Through the application of a Hellwig’s synthetic indicator of development, this study has examined the level of socioeconomic development of the EU member states. Consistent with existing studies in this area (e.g., Kuc 2012; Olczyk 2014; Grzebyk and Stec 2015), there is a significant variation in the level of development within the EU member states, as showed by the coefficients of variation (27.84 %).

The primary goal of this study is to examine the adoption of ISO 14001 certification system in the EU member states. The results show that the level of national socioeconomic development is a factor associated with the diffusion of ISO 14001 environmental management system in the EU countries. The analyses verified our hypothesis that there is a correlation between the level of national socioeconomic development and the adoption of the ISO 14001 certification. Specifically, while there was no relationship between the number of ISO 14001 certificates and the level of socioeconomic development at the national level in 2012, a weak negative correlation was observed between the increase of certification from 2011 to 2012 and the level of national socioeconomic development. The results suggest a higher interest in the ISO 14001 adoption by firms from less developed countries than that by the firms located in the highly developed states. Such a result is consistent with some existing research, e.g., by Chapple et al. (2001) who argue that ISO 14001 certificate adoption tends to be less likely when a firm becomes more powerful in terms of its level of export, market share, and profitability. They are also in line with the research by Heras-Saizarbitoria et al. (2015) which shows that the adoption of environmental management systems among the EU member states differs by environmental impacts of economic activities sectors.

The observed relationship is moreover supported by the theory of performance frontiers (Schmenner and Swink 1998). According to this theory, the best returns from investment—including environmental investments—should be expected in the early stage of implementation. After the initial stage, the positive effects of investments demand additional resources (Vastag 2000). It seems that improvements resulting from international standard certification are more substantial in less developed countries with large productivity differences among the companies, most of which function below the technological possibility frontier. In such environments, international management standards can significantly help firms move up the technological ladder, improve their productivity, and enhance their public images (Goedhuys and Sleuwaegen 2013). Therefore, the higher potential benefits accrued from the ISO 14001 adoption in less developed countries might explain the higher increase of ISO 14001 certification in these countries.

While the implementation of ISO 14001 environmental management system still remains an important tool for market success, such interests are mainly from less developed countries. Apparently, the high level of national socioeconomic development seems not translating into a higher interest in compliance with ISO 14001 certification. Less interest in ISO 14001 certification in businesses from more developed countries might be however explained by the saturation in the system adoption. And consequently, higher interest in the adoption by firms in developing countries might also be connected with the less mature certification systems. Further research needs to be conducted to compare the level of saturation in ISO 14001 certification between more and less developed countries.

## Footnotes

1 Stimulant is a variable whose high value signals a favourable situation of an object. Destimulant is a variable whose high value signals an unfavourable situation of an object (Pociecha and Zając 1989).
